# Structure-Function Relationship between Cluster Mean Defect and Sector Peripapillary Retinal Nerve Fiber Layer Thickness in Primary Open Angle Glaucoma

**DOI:** 10.1155/2022/5231545

**Published:** 2022-07-11

**Authors:** Jing Han, Wenjiu Yang, Dabo Wang, Haiqing Bai

**Affiliations:** Affiliated Hospital of Qingdao University, Qingdao, China

## Abstract

**Purpose:**

To determine the structure–function relationship between cluster mean defect (MD) offered by standard automated perimetry and corresponding sector peripapillary retinal nerve fiber layer thickness (pRNFLT) measured with optical coherence tomography (OCT) in primary open angle glaucoma (POAG).

**Method:**

39 healthy eyes (control group), 43 early POAG eyes (global MD ≤ 6 dB, early group), 30 moderate POAG eyes (global MD between 6 and 12 dB, moderate group), and 53 advanced POAG eyes (global MD > 12 dB, advanced group) underwent visual field (VF) examination with Octopus perimeter (dynamic strategy/G2 pattern) and peripapillary retinal nerve fiber layer thickness measurements with RTVue-100 FD-OCT. Spearman analysis was used to investigate the correlation between cluster MDs provided by Octopus perimeter and corresponding sector pRNFLT for the total sample and each subgroup, respectively. Then, linear (*y* = *a*+ b*x*) and curvilinear (quadratic, *y* = *a*+b*x* + c*x*^2^) regression analyses were employed to investigate the model for the cluster MD-sector pRNFLT pair with significant correlation. The strength of the relationship was characterized with correlation coefficient (*ρ*) and coefficient of determination (*R*^2^). For the cluster–sector pair that could be fitted by both models, Wilcoxon signed rank test of absolute residuals was used to compare the goodness of fit.

**Results:**

Correlation between cluster MDs and corresponding sector pRNFLT was significant for all clusters in the total sample (*ρ* values: −0.572 to 0.832, *P* < 0.001) and in the POAG group (*ρ* values: −0.551 to −0.777, *P* < 0.001). The highest *ρ* values were found for cluster-sector pair 9 and pair 3, respectively. The curvilinear (quadratic) model provided better fit for all 10 cluster-sector pairs in the total sample (*R*^2^ values: 0.431–0.687, *P* < 0.001) and in the POAG group (*R*^2^ values: 0.364–0.594, *P* < 0.01). The highest *R*^2^ values were found also for cluster–sector pair 9 and pair 3, respectively. In the control group, no significant correlation was found for any cluster–sector pair (*P* > 0.01). In the early group, correlation was significant for cluster–sector pairs 3, 8, and 9 (*ρ* values: −0.449, −0.627, and −0.815, resp., *P* < 0.01). In the moderate group, correlation was significant for pairs 2, 3, 8, and 9 (*ρ* values: −0.703, −0.556, −0.680, and −0.637, resp., *P* < 0.01). In the advanced group, correlation was significant (*P* < 0.01) for all 10 pairs (*ρ* values: −0.395 to −0.699, *P* < 0.001) except for pairs 2, 3, and 8, and the highest *ρ* value was found for pair 1. For all cluster–sector pairs with significant correlation in the early, moderate, and advanced groups, only linear model could be fitted (*P* < 0.01), except for pair 9 in the early group and pair 5 in the advanced group.

**Conclusions:**

Cluster MD of the Octopus visual field showed significant moderate-to-strong negative correlation and curvilinear (quadratic) relationship with the corresponding sector pRNFLT for POAG. This type of regional structure–function relationship varied according to the severity of POAG, and at each stage, the significantly correlated cluster–sector pairs mainly showed linear relationship. The results could provide guidance for better utilization of this regional structure–function method in the management of different stages of POAG.

## 1. Introduction

Primary open angle glaucoma (POAG) is a progressive optic neuropathy characterized by distinctive structural and functional damage. The apoptosis of retinal ganglion cells (RGCs) accompanied by loss of related axons is the common pathophysiological process of structural and functional damage. Therefore, in the course of the disease, the measurements of structure and function should be related, which is called the structure–function relationship [1, 2]. In both structural and functional examinations of glaucoma, certain variabilities and fluctuation are prevalent between individuals and repeated measures, so it is usually challenging to determine whether the disease is progressing based on only one aspect of structural or functional measurements. While by taking advantage of the structure–function relationship, clinicians can combine the structural and functional measurements to make clinical decision [1].

To date, visual field (VF) examination by standard automated perimetry (SAP) remains the gold standard for function evaluation and progression detection of POAG. Researchers using progression analysis methods of SAP need to compare individual follow-up results with the SAP database, and the statistically significant decrease (*P* < 5%) will be judged as progression. Whereas overreliance on statistical results of SAP may lead to the omission of slight progression, the mild or nonstatistically significant measurement changes may be clinically meaningful when they show correspondence or consistency with structural measurements. Therefore, exploring the structure–function relationship of POAG has always been the focus.

Among the various structural measurements, peripapillary retinal nerve fiber layer thickness (pRNFLT) by optical coherence tomography (OCT) is still the most clinically significant parameter for structure evaluation of POAG [3, 4]. Studies have shown that between VF global mean defect (MD) and average pRNFLT, there is a moderate negative and nonlinear relationship for POAG patients [5–7]. However, both average pRNFLT and MD are summary global metrics that quantify overall pRNFLT or VF sensitivity loss and reflect the average damage level. It is recognized that both structural and functional damage of POAG are manifested as typical local damage gradually developing into diffuse damage [8, 9]. Therefore, summary global metrics inevitably consider unequal spatial information and hamper the strength and nature of the structural–functional relationship [10]. In addition, some subtle localized glaucomatous progression may be masked by the analysis of summary global metrics [11, 12]. Therefore, exploring the relationship between spatial corresponding regional structural and functional metrics is of considerable importance not only for clarifying the structure–function relationship but also for improving the sensitivity and specificity of progression detection [5, 13].

Most OCT instruments can provide regional pRNFLT. In Humphrey and Octopus perimeters, which are the most widely used SAPs, there are standard methods to group the VF test points into clusters. These make it possible to investigate the relationship between spatial corresponding regional structural and functional metrics. In the Octopus perimeter, there are 59 test points in the glaucoma pattern that follow the retinal nerve fiber bundle distribution. As a result, test points corresponding to the same retinal nerve fiber bundle are automatically grouped into individual clusters. Thus, 10 clusters containing different numbers of points are formed. The mean sensitivity and mean defect of the points contained in each cluster are calculated separately, namely, cluster mean sensitivity values (cluster MSs) and cluster mean defect values (cluster MDs) [14]. Previous studies have shown strong positive and parabolic relationship between narrow sector pRNFLT and corresponding cluster MSs [15] and a moderate-to-strong negative relationship between 10 sector pRNFLT and corresponding cluster MDs [16] for overall populations, including glaucoma sufferers. However, these researchers did not further examine the relationship according to glaucoma disease severity. It is well known that, in different stages of POAG, the nature and location of the VF damage vary, and the trends in the pRNFLT change with varying degrees of VF defects [17]. Thus, the range of disease severity influences the inferred structure–function relationship [1, 18]. When using this cluster method to combine regional structure and function measurements for clinical decision-making and further research, whether the results from the overall POAG population could be applied to patients belonging to a certain stage, this needs further research.

In our study, sector pRNFLT was provided by RTVue-100 OCT. The Octopus perimeter was used to perform the VF examination and offer cluster MDs, and sector pRNFLT and cluster MD were correlated according to the distribution of retinal nerve fiber and the method used in previous studies [15, 16]. The relationship between corresponding sector pRNFLT and cluster MD was explored in the total sample and in POAG patients of different stages to improve the understanding of this structure–function relationship and to provide guidelines on the application of this method.

## 2. Methods

Our study protocol adhered to the tenets of the Declaration of Helsinki and was approved by the Institutional Review Board of the Affiliated Hospital of Qingdao University. The study was conducted in the Department of Ophthalmology at the Affiliated Hospital of Qingdao University. Informed consent was obtained from each participant before enrollment.

### 2.1. Participants and Patient Groups

The study comprised healthy and POAG subjects. Before enrollment, all subjects completed a detailed ophthalmic examination, which included a review of their medical history, corrected distance visual acuity, intraocular pressure by Goldmann tonometry, slit-lamp biomicroscopy of the anterior segment and gonioscopy, detailed fundus and optic disc inspections, Octopus G2 threshold perimetry (Haag-Streit AG, Switzerland) of the central 30 degrees VF using phases 1 and 2 of the dynamic strategy, and pRNFLT examination by RTVue-100 OCT (Optovue Inc., Fremont, CA, USA). All examinations were performed by the same experienced ophthalmologist and technician and on the same day for each participant.

The inclusion criteria consisted of being aged between 18 and 60 years old, having at least two experiences of Octopus dynamic G2 VF threshold testing before this study (for healthy eyes, two VF tests were performed on different days before enrollment), having no other eye diseases or serious systemic diseases that could affect the reliability of the results, having corrected distance visual acuity of 0.8 (Snellen equivalent 20/25) or better and refractive error within ±5.00-dioptre equivalent sphere and ±2.00-dioptre astigmatism, having corneal thickness within the range of 500 *μ*m∼550 *μ*m, having IOP within 10∼21 mmHg (with/without regular IOP-lowering drugs), having transparent ocular media, and having an open anterior chamber angle. The exclusion criteria consisted of a history of intraocular surgery or laser treatment within three months before screening, having serious systemic diseases, and being a pregnant woman or nursing mother.

If both eyes of one subject met the inclusion criteria, one eye was randomly chosen. The control group comprised 39 healthy eyes with no family history of glaucoma, normal optic nerve head appearance, normal VF, and no other sign or symptom associated with glaucoma. A total of 43 early POAG (global MD ≤ 6 dB), 30 moderate POAG (global MD between 6 and 12 dB), and 53 advanced POAG (global MD > 12 dB) eyes characterized by glaucomatous neuroretinal rim loss and reliable and reproducible VF defect typical for glaucoma (inferior and/or superior paracentral or arcuate scotomas, nasal step, hemifield defect, or generalized depression with MD worse than 2 dB) were included in the study.

### 2.2. Visual Field Testing and Determination of the Visual Field Clusters

**Figure 1:**
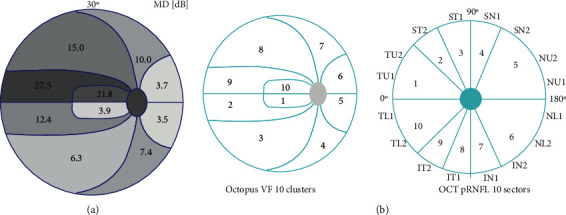
The Octopus EyeSuite software-provided 10 VF cluster MDs (a) (one advanced right eye). Spatial relationship between the manufacturer-provided 10 Octopus VF clusters and the 10 customized RTVue-100 OCT RNFL sectors (b). The spatially corresponding areas are indicated with numbers inside the graphs. The 16 22.5°-sized RNFL sectors, which are automatically provided by the OCT instrument's software, are indicated outside the nerve fiber sector graph. IN: inferonasal; IT: inferotemporal; NL: nasal lower; NU: nasal upper; SN: superonasal; ST: superotemporal; TL: temporal lower; and TU: temporal upper.

The same Octopus 900 perimeter (Haag-Streit AG, Switzerland) with G2 pattern of the central 30 degrees VF (phases 1 and 2, which provide doubled threshold determination) and dynamic strategy were applied for all examinations. Current ametropia was corrected for according to the manufacturer's recommendation. Only reproducible tests with <20% false-positive and 20% false-negative response rates were used for evaluation. The software-provided 10 VF cluster MDs were used [14] (Figure 1(a)). The clusters of the left eyes were mirrored and numbered the same as those of right eyes.

### 2.3. Optical Coherence Tomography

All participants were imaged with the RTVue-100 OCT (Optovue Inc., Fremont, CA, USA) without pupil dilation. The RTVue-100 OCT uses a near-infrared light source centered at 840 nm, with a 50 nm bandwidth, and the standard glaucoma protocol was used for pRNFLT measurements. Each optic nerve head scan consists of 12 radial lines and 6 concentric rings, which are used to create a pRNFLT map. The 3.5 mm-diameter circle composed of 920 points is derived from this map after the sample circle is adjusted to be centered on the optic disc. The measured pRNFLT is automatically given for the total circle, the superior and inferior sectors, and each of the 16 22.5°-sized sectors of the measuring circle (Figure 1(b)). The 16 sectors are numbered in sequence from the temporal side of the horizontal meridian (clockwise for the right eye and anticlockwise for the left eye). Image quality was carefully checked after each image acquisition, and all images of insufficient quality or with any artifact were rejected and reacquired. Only images with signal strength index >40 were used.

The spatial corresponding relationship between the manufacturer-provided 10 VF clusters and the 10 customized RTVue-100 OCT RNFL sectors as used in the current investigation is seen in Figure 1(b). For 1, 5, 6, and 10 pRNFLT sectors, which comprised two or three 22.5°-sized sectors, the average of the 2 or 3 sector pRNFLT values was calculated.

### 2.4. Statistics

The SPSS software (SPSS for Windows, version 19.0; SPSS Inc., Chicago, IL, USA) was used for statistical analysis. The normality of distribution of the study sample was assessed with the Shapiro–Wilk test. Given that the data of some subgroups were not normal distribution, Spearman analysis was used to investigate the correlation between global MD and global mean pRNFLT and corresponding cluster MDs and sector pRNFLT values for the total sample, the POAG group, and each subgroup. A *ρ* value <0.2 represented a negligible relationship; between 0.21 and 0.39 a weak relationship; between 0.4 and 0.59 a moderate relationship; between 0.6 and 0.79 a strong relationship, and over 0.8 a very strong correlation. Then, linear (*y* = *a*+b*x*) and curvilinear (quadratic *y* = *a*+b*x* + c*x*^2^) regression analyses were employed to investigate the model of relationship for each structure–function pair with significant correlation. The strength of the relationship was characterized with coefficient of determination (*R*^2^), which indicates how well-observed outcomes are replicated by the model, as the proportion of total variation in outcomes explained by the model. The Wilcoxon signed rank test of absolute residuals was used to compare the goodness of fit between the two models when both models could be fitted. The Octopus software-provided dB values for global MD and cluster MD values were used. VF parameters were the dependent variables, and pRNFLT (*μ*m) was the independent variable. *P* values of <0.01 were considered statistically significant.

## 3. Results

The demographics of the participants are shown in Table 1. There were no differences in the mean age of the different groups (*P* > 0.01). From the control group to the advanced POAG group, as absolute global MD values increased, global pRNFLT decreased, and the differences were significant (*P* < 0.01). For all POAG individuals, the mean global MD was 12.25 ± 7.92 dB, and the average global mean pRNFLT was 60.87 ± 19.30 *μ*m (Table 1).

The correlation between the corresponding cluster MD and sector pRNFLT values was significant for all 10 cluster–sector pairs (*ρ* values: −0.572 to −0.832, *P* < 0.001) in the total sample which including healthy and POAG eyes, and the strongest *ρ* value was observed for the cluster–sector pair 9. For the POAG group, correlation was significant for all 10 cluster–sector pairs (*ρ* values: −0.551 to −0.777, *P* < 0.001), and the strongest correlation was observed for cluster–sector pair 3 (Table 2). In the control group, no significant correlation was found for any cluster–sector pair (*P* > 0.01). In the early group, correlation was significant for cluster–sector pairs 3, 8, and 9 (*ρ* values: −0.449, −0.627, and −0.815, resp., *P* < 0.01). In the moderate group, correlation was significant for cluster–sector pairs 2, 3, 8, and 9 (*ρ* values: −0.700, −0.574, −0.681, and −0.612, resp., *P* < 0.01). In the advanced group, correlation was significant (*P* < 0.01) for all 10 cluster–sector pairs except for 2, 3, and 8. A strong negative relationship was seen for cluster–sector pairs 1, 10, and 4, (*ρ* values: −0.699, −0.613, and −0.611, resp.); a moderate negative relationship was seen for pairs 6, 7, and 9 (*ρ* values: −0.559, −0.536, and −0.406, resp.); and for pair 5, the negative relationship was weak (*ρ* value: −0.395) (Table 2).

**Figure 2:**
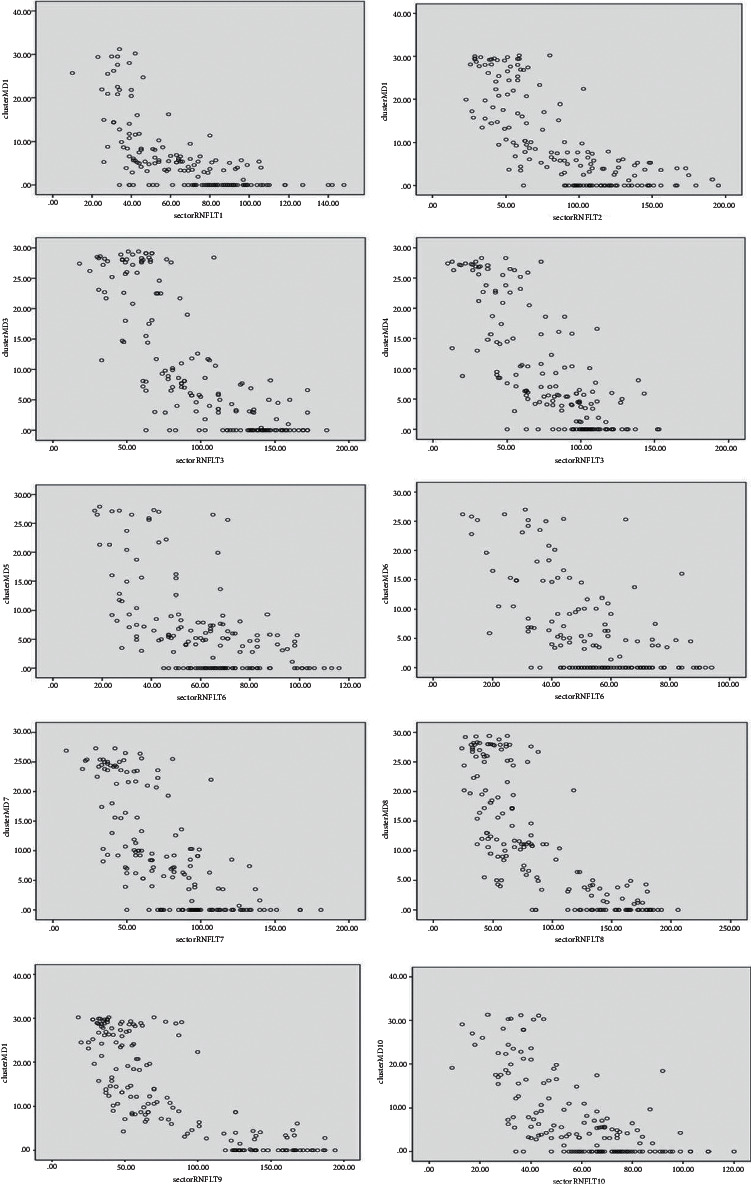
Scatter plots of sector retinal nerve fiber layer thickness (RNFLT, *μ*m) and cluster mean defect (cluster MD, dB) for 10 cluster-sector pairs in the total sample (*n* = 165).

**Figure 3:**
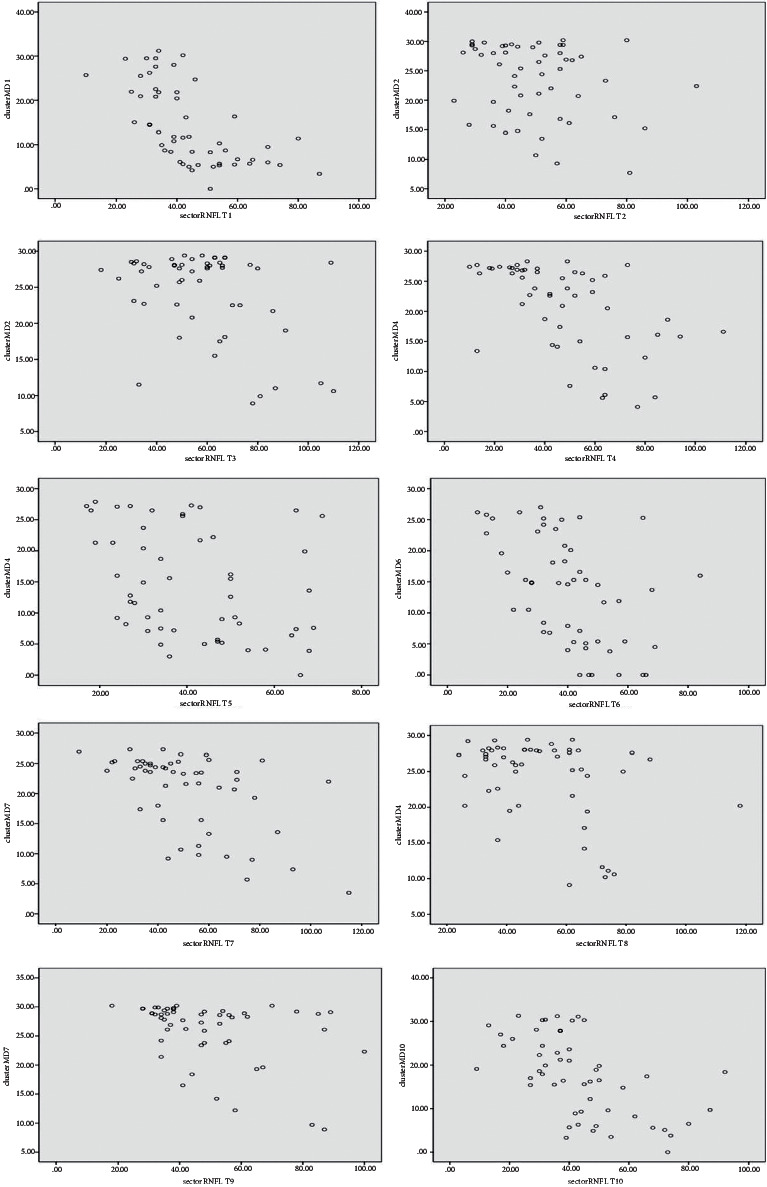
Scatter plots of sector peripapillary retinal nerve fiber layer thickness (pRNFLT, *μ*m) and cluster mean defect (cluster MD, dB) for 10 cluster-sector pairs in the advanced group (*n* = 53).

In the total sample, curvilinear (quadratic) model provided a better fit for all 10 cluster–sector pairs (*R*^2^ values: 0.431–0.687, *P* < 0.001), and the strongest *R*^2^ value was observed for cluster–sector pair 9. For the POAG group, the curvilinear (quadratic) model provided a better fit for all 10 cluster–sector pairs (*R*^2^ values: 0.364–0.594, *P* < 0.01), and the strongest *R*^2^ value was observed for cluster–sector pair 3 (Table 3). For all cluster–sector pairs with significant correlation in the early, moderate, and advanced groups, only a linear relationship could be fitted (*P* < 0.01), except for cluster–sector pair 9 in the early group, for which both linear and curvilinear (quadratic) models were established and the *R*^2^ value of the latter was greater than that of the former (*R*^2^ values: 0.698 and 0.580, resp., *P* < 0.01), and cluster–sector pair 5 in the advanced group, for which neither of the two models could be fitted (*P* > 0.01). The highest *R*^2^ values were seen for cluster–sector pair 9 in the early group, cluster–sector pair 8 in the moderate group, and cluster–sector pair 1 in the advanced group (Table 3). The scatter plots for each cluster–sector pair in the total sample and in the advanced group are shown in Figures 2 and 3.

Between global MD and global mean pRNFLT, correlation was significant in the total sample and in the POAG subgroup (*ρ* values: −0.842 and −0.809, resp., *P* < 0.001), and the curvilinear (quadratic) model provided the better fit (*R*^2^ values: 0.751 and 0.638, resp., *P* < 0.001). In the control group and early group, no significant correlation was found (*P* > 0.01). In the moderate and advanced groups, the correlation was significant (*P* < 0.01), and the *ρ* values were −0.522 and −0.502, respectively. Only a linear curve could be fitted for the moderate group (*R*^2^ value: 0.289, *P*=0.0022), and for the advanced group, the *R*^2^ value of the curvilinear (quadratic) model was greater than that of the linear curve (*R*^2^ values: 0.275 and 0.167, resp., *P* < 0.01).

## 4. Discussion

To date, no optimum and universally accepted criteria for the detection of progression for POAG have been established for any structure and function analysis tools [9]. Excessive reliance on statistical tools and statistical boundaries in various software may lead to the omission of slight progression. The unique pathophysiological characteristics of glaucoma determine that the combination of structural and functional measurements will optimize the sensitivity and specificity of the detection of progression. Our research provides guidance on combining sector pRNFLT by OCT and VF cluster MD.

The purpose of our study was to determine the structure–function relationship between VF cluster MDs and corresponding sector pRNFLT for POAG. We found moderate-to-strong and even very strong correlation for all 10 cluster MD-sector pRNFLT pairs both for the overall population comprising healthy and POAG eyes and for the POAG group, and compared with linear regression, the curvilinear (quadratic) relationship provided the better fit, while at each stage, the significantly correlated cluster-sector pairs mainly showed linear relationship. Clinicians can utilize this relationship in progression monitoring because when the same decreases occur in the corresponding region in VF and pRNFLT, even if those decreases are extremely slight and cannot meet the statistical criteria for judging progression, they may still be meaningful as an indicator for progression. However, when using this method, we must keep in mind that the relationship varied according to the severity of the disease; the pairs with a stronger relationship in each stage were different, and in certain stages for some cluster-sector pairs, the correlation was not significant. This was because of the unequal variability in normal pRNFLT and VF measurements, the limitations in the dynamic range of structural–functional tests in cases with severe damage, and the different characteristics of POAG damage in each stage.

Our results on the relationship between the corresponding cluster MD and sector pRNFLT values in the total sample were in agreement with the previous study [16]. Hollo et al. found significant, moderate-to-strong negative correlations for all 10 cluster–sector pairs, and the strongest relationship was also found for the inferotemporal pRNFLT sector superior and superior paracentral cluster pair (pair 9) [16]. However, in their study, in the glaucoma subgroup, a significant negative correlation was seen only for cluster–sector pair 9. In our study, for all POAG individuals, there were moderate-to-strong relationships for all 10 cluster MD-sector pRNFLT pairs. The differences may be caused by the different severity of the glaucoma individuals enrolled in the studies. Our POAG subjects came from all stages, including advanced patients, and the mean global MD was 12.25 ± 7.92 dB, whereas in their study, the glaucoma subjects were early-to-moderate, and the mean global MD was 5.7 dB. In the early and moderate groups of our study, indeed only cluster-sector pairs 2, 3, 8, and 9 had significant correlation. These results coincide with the usual clinical findings in glaucoma; the inferotemporal and superotemporal sectors are more frequently damaged in early and moderate stages, and they match with superonasal and inferonasal paracentral cluster defects in the VF. For the regions with no damage or very slight damage in early-to-moderate stage, the morphological variability is great, while the dynamic range of the cluster MD is limited. For example, the MD of cluster 1 was 0 (0, 3.9) dB in cluster-sector pair 1 without significant correlation in the early stage, in which 53.5% of cluster MD values were 0, while the pRNFLT of sector 1 was 79 (61, 93) *μ*m. The asymmetry of dynamic ranges causes these cluster–sector pairs not to have significant correlation. This was further confirmed in the normal population. Consistent with previous studies [15, 16], in the normal group, there was no significant correlation between structural and functional measurements for either global metrics or any cluster–sector pair. Taking global metrics as examples, the normal dynamic range of pRNFLT in our study ranges from 95 *μ*m to 116 *μ*m, while the normal range of global MD was only −2 to +2 dB. Similarly, for those cluster–sector pairs without significant correlation in the early and moderate subgroup, the same dynamic range asymmetries existed between sector pRNFLT and cluster MD. For example, in cluster–sector pair 1, without significant correlation in the moderate group, the dynamic range of its sector pRNFLT ranges from 26 to 106 *μ*m (the minimum value in the advanced group was 25 *μ*m). In contrast, the range of its cluster MD was only 0 to +8.8 dB (the maximum value in the advanced group was 31.2 dB).

In general, the greater the damage in the VF, the stronger the correlations between OCT and SAP findings [19]. For advanced POAG individuals, the region affected by glaucoma damage expanded, and the damage gradually changed from local damage to diffuse damage. The cluster–sector pairs that were originally unrelated showed significant correlation in our advanced group. This indicates that once glaucomatous damage occurred in these regions that had not been damaged in the early-to-moderate stage, there was a significant correlation between their cluster MDs and corresponding sector pRNFLT. The two pairs with the strongest correlation were pairs 1 and 10, that is, the nerve fiber bundles from the superior macula, which is known to be resistant to glaucomatous structural damage and usually remains intact until the final stages of the disease are attained [20], and they match with the central VF, which is frequently involved in advanced glaucoma [21, 22]. Our findings were consistent with clinical findings. In clinical settings, the VF in advanced glaucoma is mostly manifested as a tubular VF. However, the originally correlated pairs 2, 3, and 8 no longer showed significant correlation. It can be seen from the scatter spots that these regions, which had been damaged in the earlier stage, were severely damaged in the later stage. Once the cluster MD reached the limit value of 30 dB, even though the pRNFLT was further thinned, the cluster MD would no longer have correspondingly deteriorated. These results suggest that for the cluster whose cluster MD was close to the limit value (30 dB), it was no longer correlated with the corresponding pRNFLT. Previous studies suggested that there was no significant correlation between global MD and pRNFLT in advanced glaucoma, mainly owing to the floor effect of pRNFLT [23]. However, in our study, we found a substantial correlation between most cluster MDs and corresponding pRNFLT for advanced POAG. This suggests that the regional structure–function relationship based on cluster MD was more valuable in detecting progression for advanced POAG, especially in the central clusters. We believe that the averaging process of the global indices masked the real relationship of the regional indices. Interestingly, the scatter plots showed that in the advanced group, some cluster MDs had no significant correlation with the corresponding sector pRNFLT, mainly because the cluster MDs reached the limit value. Whether cluster MDs reach the measurement platform earlier than regional pRNFLT requires further follow-up of the advanced individuals. In summary, we suggest that for advanced POAG, clinicians should pay more attention to the cluster MD-sector pRNFLT pairs with relatively minor damage and more close correlation to obtain more information about disease progression.

In Naghizadeh et al.'s report, a significant parabolic relationship was found between the 16 Octopus VF cluster MSs and the corresponding 16 pRNFLT sectors measured with RTVue OCT for a population comprising normal, ocular hypertensive, and glaucoma eyes, and for the glaucoma subgroup [15]. In our study, for the total population and for the POAG subgroup, curvilinear (quadratic) model also provided the better fit for all 10 cluster MD-sector pRNFLT pairs. To date, there is no report on the model of the relationship between cluster MD and sector pRNFLT in POAG. The curvilinear relationship between structural and perimetric measurements may be caused by the wide variability in normal structure and the limitations of the dynamic range of structural measurements in advanced POAG. Previous researchers have found large changes in the average pRNFLT in eyes with early damage corresponding to small changes in the global MD; however, for eyes with an advanced stage of the disease, large changes in the global MD only corresponded to small changes or no changes in the average pRNFLT [24]. Combined with the scatter plots, we found that the aforementioned phenomenon between the global structural and functional metrics was also applicable to the regional metrics, so the better relationship model was curvilinear (quadratic) model. However, for most of the cluster-sector pairs with significant correlation in the early, moderate, and advanced groups, only a linear relationship could be fitted. This indicates that in each specific disease stage, there was a linear correspondence between cluster MD and sector pRNFLT. These results are valuable for utilizing the regional structure and function parameters to monitor the progression of glaucoma.

Our study has limitations. Compared to previous studies, no ocular hypertensive eyes were included. In previous studies on ocular hypertensive eyes, there was no relationship between structural and functional metrics. Second, to ensure the credibility of VF examination, we had higher requirements for central vision, so we did not include advanced patients whose central vision was seriously impaired. For extremely advanced patients, the relationship between cluster MD and sector pRNFLT needs further research. In our advanced group, for the pairs whose cluster MD was close to the limit value, the correlation was not significant.

In conclusion, significant moderate-to-strong and even very strong negative correlations and curvilinear (quadratic) relationships were found between the 10 Octopus VF cluster MD values and the corresponding pRNFLT sectors measured with RTVue OCT for a population comprising normal and POAG eyes and for the POAG subgroup. Nevertheless, the relationship varied according to the severity of the disease, and the pairs with a stronger relationship in each stage were different. In each stage, for the corresponding cluster and sector regions suffering glaucomatous damage, they showed significant linear correlation. The study will help to clarify the regional structure–function relationship in POAG, and the results show that this method can be applied for structure-function-based clinical decisions in progression detection. That is, when the corresponding cluster MDs and sector pRNFLT progress simultaneously, even if these changes have no statistical significance in the software, clinicians should be alert and combine them with other examination results or increase the frequency of examination to detect progression in time. This method may be more sensitive than only relying on global indices and statistical significance.

## Figures and Tables

**Table 1:** Demographics of the participants.

	Control group		All POAG individuals		Early group		Moderate group		Advanced group		*P*
	Mean/median	SD/quartile	Mean/median	SD/quartile	Mean/median	SD/quartile	Mean/median	SD/quartile	Mean/median	SD/quartile	
No. of eyes	39		126		43		30		53		
Age (years)	40.28	11.12	40.84	10.32	41.58	11.98	39.43	8.26	41.51	10.39	0.538
Gender (female/male)	15/24		39/87		13/30		12/18		14/39		0.256
Global MD (dB)	0.67	0.54	12.25	7.92	4.40	1.06	9.55	1.66	20.58	4.41	≤0.001
Global pRNFLT (*μ*m)	103.51	6.41	60.87	19.30	79.09	14.55	62.30	13.17	45.26	10.17	≤0.001
Cluster MD 1	0	0,0	5.4	0,10	0	0,3.9	4.7	0,5.8	11.6	6.1,21.9	≤0.01
Cluster MD 2	0	0,0	9.9	4.7,23.5	4.0	2.5,6.0	7.8	5.3,11.1	25.4	17.9,29.1	≤0.001
Cluster MD 3	0	0,0	9.9	5,26.1	4.0	0,6.5	8.1	5.9,12.0	27.6	22.1,28.3	≤0.001
Sector pRNFLT 3	143	134,157	80	60,110.5	112	93,138	87	69.8,107.5	58	46.56.7	≤0.001
Sector pRNFLT 4	107	101,121	67	44,94	95	75,106	75	48.5,91	46	31,63.5	≤0.001
Sector pRNFLT 5	71	65,88	56	41,70	71	62,84	57	47,70	39	29,50.5	≤0.001
Sector pRNFLT 6	63	55,71	50.5	40,61.3	59	53,73	49.5	42.8,59.2	40	30.5,49	≤0.001
Sector pRNFLT 7	113	98,133	64.5	46,88	88	67,96	63.5	49,82.3	48	36,62	≤0.001
Sector pRNFLT 8	164	143,179	61.5	45,84	86	57,132	67	48.8,84	47	36.5,64.5	≤0.001
Sector pRNFLT 9	145	129,167	55	39,80	75	54,138	55	43.5,67.8	47	35,57.5	≤0.001
Sector pRNFLT10	76	66,87	51	37,68	67	55,74	53.5	38.5,67.8	40	31,50	≤0.001

MD: mean defect; pRNFLT: peripapillary retinal nerve fiber layer thickness.

**Table 2:** Structure–function correlation results between sector pRNFLT and corresponding cluster MD in each group (*P* < 0.01).

Cluster–sector pair	The total sample		POAG group		Early group		Moderate group		Advanced group	
	*ρ*	*P*	*ρ*	*P*	*ρ*	*P*	*ρ*	*P*	*ρ*	*P*
Cluster–sector pair 1	−0.732	≤0.001	−0.671	≤0.001	−0.243	0.117	−0.310	0.096	−0.699	≤0.001
Cluster–sector pair 2	−0.787	≤0.001	−0.763	≤0.001	−0.315	0.040	−0.703	≤0.001	−0.196	0.159
Cluster–sector pair 3	−0.821	≤0.001	−0.777	≤0.001	−0.449	0.003	−0.556	0.001	−0.241	0.082
Cluster–sector pair 4	−0.771	≤0.001	−0.710	≤0.001	−0.252	0.103	−0.325	0.080	−0.611	≤0.001
Cluster–sector pair 5	−0.572	≤0.001	−0.551	≤0.001	−0.007	0.963	−0.073	0.700	−0.395	0.003
Cluster–sector pair 6	−0.581	≤0.001	−0.581	≤0.001	−0.119	0.448	−0.090	0.635	−0.559	≤0.001
Cluster–sector pair 7	−0.757	≤0.001	−0.674	≤0.001	−0.168	0.280	−0.339	0.067	−0.536	≤0.001
Cluster–sector pair 8	−0.825	≤0.001	−0.693	≤0.001	−0.627	≤0.001	−0.680	≤0.001	−0.325	0.017
Cluster–sector pair 9	−0.832	≤0.001	−0.686	≤0.001	−0.815	≤0.001	−0.637	≤0.001	−0.406	0.003
Cluster–sector pair 10	−0.695	≤0.001	−0.651	≤0.001	−0.321	0.036	−0.439	0.015	−0.613	≤0.001

POAG group: including early, moderate, and advanced eyes.

**Table 3:** The regression results of the cluster–sector pairs with significant correlation in each subgroup (*P* < 0.01).

Structure–function pair	Linear regression		Curvilinear regression	
	*R* ^2^	*P*	*R* ^2^	*P*
Cluster–sector pair 3 in early group	0.186	0.004	0.233	0.127
Cluster–sector pair 8 in early group	0.310	≤0.001	0.378	0.043
Cluster–sector pair 9 in early group	0.580	≤0.001	0.698	≤0.001
Cluster–sector pair 2 in moderate group	0.329	0.001	0.360	0.270
Cluster–sector pair 3 in moderate group	0.337	0.001	0.362	0.311
Cluster–sector pair 8 in moderate group	0.497	≤0.001	0.497	0.952
Cluster–sector pair 9 in moderate group	0.402	≤0.001	0.475	0.063
Cluster–sector pair 1 in advanced group	0.393	≤0.001	0.438	0.052
Cluster–sector pair 4 in advanced group	0.317	≤0.001	0.322	0.536
Cluster–sector pair 5 in advanced group	0.115	0.013	0.169	0.079
Cluster–sector pair 6 in advanced group	0.244	≤0.001	0.304	0.042
Cluster–sector pair 7 in advanced group	0.304	≤0.001	0.304	0.950
Cluster–sector pair 9 in advanced group	0.153	0.004	0.164	0.012
Cluster–sector pair 10 in advanced group	0.339	≤0.001	0.355	0.267

## Data Availability

The data supporting the results of the current article are available from the corresponding author.
